# Shell colour diversification induced by ecological release: A shift in natural selection after a migration event

**DOI:** 10.1002/ece3.8080

**Published:** 2021-10-19

**Authors:** Shun Ito, Takahiro Hirano, Satoshi Chiba, Junji Konuma

**Affiliations:** ^1^ Graduate School of Life Science Tohoku University Miyagi Japan; ^2^ Center for Northeast Asian Studies Tohoku University Miyagi Japan; ^3^ Department of Biology, Faculty of Science Toho University Chiba Japan

**Keywords:** colour diversification, disruptive selection, ecological release, land snail, mammal predator, mark–recapture, stabilizing selection, trail camera

## Abstract

Ecological release is often attributed to the rapid adaptive diversification of phenotypic traits. However, it is not well understood how natural selection changes its strength and direction through the process of ecological release. Herein, we demonstrated how shell colour of the Japanese land snail *Euhadra peliomphala simodae* has diversified via a shift in natural selection due to ecological release after migration from the mainland to an island. This snail's shell colour diversified on the island due to disruptive selection after migration from the mainland. We used trail camera traps to identify the cause of natural selection on both the mainland and the island. We then conducted a mark–recapture experiment while collecting microhabitat use data. In total, we captured and marked around 1,700 snails on the mainland, some of which were preyed upon by an unknown predator. The trail camera traps showed that the predator is the large Japanese field mouse *Apodemus speciosus*, and the predatory frequency was higher on the mainland than on the island. However, this predation did not correlate with shell colour. Microhabitat use on the island was more extensive than on the mainland, with snails on the island using both ground and arboreal microhabitats. A Bayesian estimation showed that the stabilizing selection on shell colour came from factors other than predation. Our results suggest that the course of natural selection was modified due to ecological release after migration from the mainland, explaining one cause of the phenotypic diversification.

## Graphical abstract

Our study demonstrates how snails' shell colour has diversified through the process of ecological release. We clarified that predation modified the natural selection via the microhabitat shift although the selection was caused from other factors.

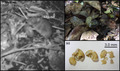

## 1 INTRODUCTION

Ecological release is a niche expansion when an interspecific interaction is reduced or removed (Herrmann et al., 2021; Stroud & Losos, 2016). Further, ecological release often has the potential for the rapid adaptive diversification of phenotypic traits. This phenomenon is called character release, which increases phenotypic variation or shifts the phenotypic mean (Herrmann et al., 2021; MacArthur & Wilson, 1967). For ecological and character release, natural selection plays a primary role in favoring specific traits according to an adaptive optimum (Schluter, 2000). For example, in the process of ecological release, the distribution of an adaptive trait should shift and diversify according to the divergent natural selection (Nosil, 2012; Schluter, 2000). In contrast, stabilizing selection works to keep the trait near a specific value in an ancestral population (Lahti et al., 2009; Schluter, 2000). However, despite these substantial observations, it has often been unclear how natural selection changes its strength and direction through the process of ecological release (Herrmann et al., 2021; Yoder et al., 2010).

The mainland–island system is ideal for studying this problem because natural selection can be rapidly modified, especially after migrating to an adjacent island (Schluter, 2000; Stroud & Losos, 2016). Species composition is lower on islands than on a mainland, and island species tend to be released from predators and competitors (Stroud & Losos, 2016). These releases change the strength and direction of natural selection on the islands (Schluter, 2000; Yoder et al., 2010). The Izu Peninsula and Islands of Japan, which were formed independently from volcanic activity less than five million years ago (Kaneoka et al., 1970; Tani et al., 2011), provide an opportunity to demonstrate how ecological release causes a shift in selection pressure. The mammalian and reptile species compositions differ between the different islands (Hasegawa, 2003; Hasegawa & Moriguchi, 1989), causing the predators and prey of these animals to evolve in unique ways on the islands. For example, the feeding behavior of the Japanese field mouse *Apodemus speciosus* shifted after migration to the islands (Takechi & Hayashi, 2012). Moreover, in the case of Okada's five‐lined skink *Plestiodon latiscutatus*, their phenotypic traits can be determined by different selective pressures from island‐specific predators (Brandley et al., 2014; Landry Yuan et al., 2021). Hence, other animals such as land snails on an island may also have been released from the numerous predators on the mainland.

Land snails on islands have presented with cases of phenotypic divergences such as in morphology and shell colour (Chiba, 1999a; Chiba & Davison, 2007; Parent & Crespi, 2006, 2009). Several studies have demonstrated that interspecific competition should trigger phenotypic divergence (Chiba, 1996, 1999b; Chiba & Davison, 2007; Kimura & Chiba, 2010). In contrast to this, predators such as mammals and birds have regulated the snails' traits (Barker, 2004; Chiba, 2007). Furthermore, environmental conditions have also been a cause of trait divergence (Kraemer et al., 2019). Overall, these factors may determine the shape and strength of natural selection, and sometimes, it promotes phenotypic diversification (Hayashi & Chiba, 2004; Ito & Konuma, 2020; Kraemer et al., 2019). In many cases, studies have focused on why an island's snails have diversified phenotypes. Still, there are almost no studies on how the snails' phenotypes became diversified by focusing on both mainland ancestral and island populations.

The shell colour diversification of snails has been studied in the Japanese land snail *Euhadra peliomphala simodae* (Hayashi & Chiba, 2000, 2004; Ito & Konuma, 2020). This snail inhabits the Izu Peninsula on mainland Japan and several of the Izu Islands. The mainland populations tend to have bright shells, while colour diversification from bright to dark is observed in the peripheral islands' sympatric populations (Hayashi & Chiba, 2004; Ito & Konuma, 2020). Previous molecular phylogenetic studies have demonstrated that the mainland population is ancestral and migrated to the islands (Hayashi & Chiba, 2000, 2004), after which shell colour diversification rapidly occurred according to natural selection (Hayashi & Chiba, 2004; Ito & Konuma, 2020). It has also been suggested that the strength of shell colour diversity varies between the islands due to differences in selection pressure (Hayashi & Chiba, 2004). For example, on Niijima Island, where shell colour diversity is the highest, strong disruptive selection has worked on the snails' shell colour (Hayashi & Chiba, 2004; Ito & Konuma, 2020). Although different acts of natural selection have been assumed, it is unknown what selection pressure acted on the mainland population. Furthermore, whether there are differences in the strength and direction of natural selection between the mainland and islands is also unknown, or why this shift occurred if there is a difference.

This study examined what species prey on the snails using trail camera traps, the differences in predation effects, and microhabitat use by focusing on the mainland and island populations. We then conducted a mark–recapture experiment with *E. p. simodae* on the mainland and estimated the natural selection pressures from predation and other factors. Finally, we compared the estimated selection pressures between the mainland and a peripheral island, Niijima Island, where we made similar estimates in a previous study (Ito & Konuma, 2020).

## 2 MATERIALS AND METHODS

### 2.1 Mark–recapture experiments

**FIGURE 1:**
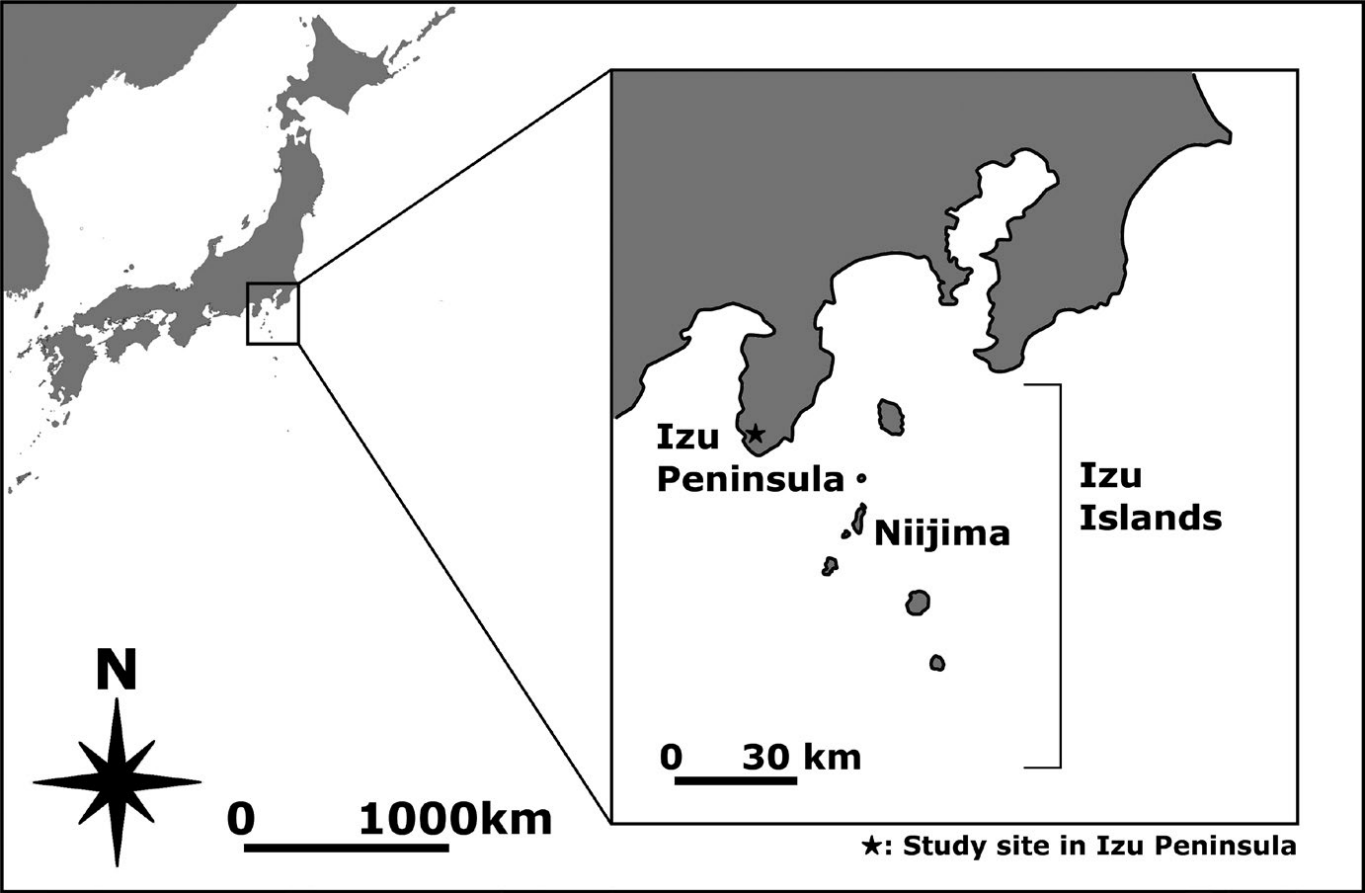
Maps of the study sites on the Izu Peninsula. *Euhadra peliomphala simodae* is found on the Izu Peninsula (mainland) and on the Izu Islands including Niijima Island. The survey was conducted in both the Izu Peninsula and the Niijima Island

We chose the Izu Peninsula and Niijima Island as sites to study *E. p. simodae* populations on the mainland and an island, respectively (Figure 1). According to a previous study (Hayashi & Chiba, 2004), *E. p. simodae* distributed in the southern part of the Izu Peninsula migrated to the Izu Islands, including Niijima Island, and then, their shell colour diversified. We selected a snail population in Minamiizu‐Cho on the Izu Peninsula as a mainland ancestral population and a population on Mount Miyatsuka on Niijima Island as an island descendent population. Both sites were narrow forest areas about 1 km long.

We conducted mark–recapture surveys every month on the mainland (February 2019 to February 2020) and island (March 2017 to October 2018). The details of the mark–recapture approach are described in a previous report (Ito & Konuma, 2020), so we include only an overview here. Each survey was conducted for 2–5 days. When we captured the snails, we recorded their growth stage, geographic location, and shell colour. The growth stage (juvenile or adult) was identified based on the shell lip. The microhabitat of each snail was also recorded as one of three categories: (a) hibernation, (b) ground‐dwelling, or (c) arboreal. It is only when snails are in hibernation that they use the ground under the leaf litter. The locations where we captured the snails were recorded using mobile GPS devices (eTrex LEGEND HCx, Garmin Ltd., Schaffhausen, Switzerland, on the mainland and a Garmin eTrex 10J on the island).

The shell colour was quantified using the RGB values of photographs of the snail shells, which were taken using a digital single‐lens reflex camera (EOS Kiss X7; Canon, Tokyo, Japan) with an EF‐S 60‐mm f2.8 USM macro lens (Canon) under a ring light in a light‐blocking box. On a computer monitor, a square area of shell close to the shell aperture was cropped using the software Photos for Mac OS X (Apple Inc., Cupertino, CA, USA). One thousand pixels within the cropped images were randomly chosen, and the RGB values of those pixels were automatically measured and averaged using Python 3 code.

The luminance value *Y* of the shell colour was calculated as *Y* = 0.299*R* + 0.587*G* + 0.114*B* (Schlick, 1995). The luminance value *Y*′ of the grayscale image (Kodak Color Grey Scale; Eastman Kodak Co., Rochester, NY, USA), photographed together with the shell, was measured in the same way. The ratio *Y*/*Y*′ was calculated to correct minor differences caused by light conditions and then standardized. A higher *Y*/*Y*′ value implies a brighter shell colour, whereas a lower *Y*/*Y*′ value implies a darker shell colour.

The captured snails were marked on the shell by gluing on coloured plastic beads and scarifying numbers on them (Henrya & Jarne, 2007). They were then released to the locations where they were caught. This study was conducted according to the animal experimentation regulations of Tohoku University and Toho University.

### 2.2 Measuring predation pressure and the identification of predators

The modes that predators use to attack gastropods are classified as shell entry, shell crushing, shell boring, and whole‐shell swallowing (DeWitt & Robinson, 2000; Vermeij, 1979). Some recovered dead snails showed evidence of shell crushing, so we considered them to have died from predator attacks rather than old age. We compared the number of predated snails between the mainland and the island using Fisher's exact test. We also analyzed the data with a generalized linear mixed model (GLMM) to examine whether the number of predations was associated with shell colour and/or growth stage. Predation (1) and nonpredation (0) were used as binary response variables, while the shell colour, growth stage (adults 1; juvenile 0), and their interaction were used as fixed effects. The month when the dead snails were recovered was used as a random effect. The “lme4” package (Bates et al., 2007) was used with logit as a link function in the statistical package R (R Core Team, 2020).

To identify the snails' predators, we conducted camera trap experiments at the mainland study site from December 2019 to July 2020 and at the island study site from April 2017 to February 2018. At these study sites, snails were secured to the ground with 5‐cm‐long strings (Daiso Ltd., Hiroshima, Japan; diameter 0.2 mm) and monitored using trail cameras (TROPHYCAM HD3 ESSENTIAL, Bushnell Ltd., Kansas, USA). Each camera was attached to a tree or on a tripod and operated on a motion‐trigger system. Each camera automatically recorded videos when an animal entered its sensor. Four and five camera traps were set up at the mainland and island study sites, respectively. We identified the predators from these videos in our laboratory.

### 2.3 Comparing microhabitat use between juvenile and adult, and the mainland and island

We conducted Fisher's exact test to analyze differences in microhabitat use. We removed the records of those in hibernation from all analyses, because whether a juvenile or adult, they were under the leaf litter when in hibernation. The analyses were compared for all combinations of island and mainland, juvenile, and adult. We corrected the *p*‐value for post hoc multiple comparisons by controlling the false discovery rate α (Benjamini & Hochberg, 1995), which was set to 0.05 in our study. Moreover, we performed the GLMM on juveniles and adults on the mainland and island to elucidate the relationship between microhabitat use and shell colour. The use of arboreal (1) and ground (0) microhabitats was used as binary response variables, while shell colour was used as a fixed effect. The month when the microhabitat use was recorded, the individual ID, was used as a random effect. We conducted these analyses on R (R Core Team, 2020). For the GLMM, “lme4” (Bates et al., 2007) was used with logit as a link function.

### 2.4 Modeling the natural selection of shell colour on the mainland

In a previous study (Ito & Konuma, 2020), we estimated the natural selection on an island, so in this study, we estimated it on the mainland. We also used the survival rate as a fitness index in the previous study (Ito & Konuma, 2020). First, second‐order polynomial regression was used to estimate the selection gradients with Equation 1 (Blows & Brooks, 2003; Gimenez et al., 2009; Lande & Arnold, 1983). To visualize the survival rate according to shell colour, we used a penalized spline regression (P‐spline) with Equation 2 (Gimenez, Covas, et al., 2006; Gimenez, Crainiceanu, et al., 2006).

(1) $$\log it\left(S_{i,t}\right)=\mu +\beta \cdot col_{i}+\frac{1}{2}\cdot \gamma \cdot col_{i}^{2}+\varepsilon_{i}+B_{t}$$

(2) $$\begin{array}{c} \log it\left(S_{i,t}\right)=f\left(col_{i}\right)+\varepsilon_{i}+B_{t} \end{array}$$

$$f\left(col_{i}\right)=\mu +\beta_{1}\cdot col_{i}+\cdots +\beta_{p}\cdot col_{i}^{P}+\sum_{k=1}^{K}b_{k}{\left(col_{i}‐\kappa_{k}\right)}_{+}^{P}$$

*t* = 1, 2, …, 12; *i* = 1, 2, …, 1,714 (in the Izu Peninsula).

In these equations, logit(*x*) means log[*x*/(1 − *x*)]; μ is the overall average for the survival rate on the logit scale; *β* is the linear selection gradient, which represents the strength and direction of directional selection; *γ* is the nonlinear selection gradient and represents the strength of disruptive selection when *γ* > 0 or that of stabilizing selection when *γ* < 0; *ϵ_i_* represents the individual heterogeneity, which follows a normal distribution with mean 0 and variance $\sigma_{\varepsilon }^{2}$ (Royle, 2008); *B_t_* is the monthly effects on the logit scale for month *t*; *P* is the degree of freedom in the P‐spline, set to *P* = 3 in this study; ${\left(col_{i}‐\kappa_{k}\right)}_{+}^{P}$ is either ${\left(col_{i}‐\kappa_{k}\right)}^{P}$ when $(col_{i}‐\kappa_{k})\geq 0$ or 0 if not; $\kappa_{k}$ represents *k's* fixed knots, with $\kappa_{1}<\kappa_{2}<\cdots <\kappa_{k}$. The number of knots *K* is decided according to $K=\min\left(\frac{I}{4},\;35\right)$, so we set it to *K* = 35 (Ruppert, 2002). We calculated *k*/(*K* + 1) quantiles using all shell colour values, with *k* varying between 1 and 35 (Ruppert, 2002). We assumed that the coefficient *b_k_* of ${\left(col_{i}‐\kappa_{k}\right)}_{+}^{P}$ follows a normal distribution with a mean of 0 and variance $\sigma_{b}^{2}$ (Gimenez, Bonner, et al., 2009). The survival rate of this species shows monthly fluctuations (Ito & Konuma, 2020), so we considered the monthly effects *t* (*t* = 1, 2, …, 12).

We constructed a multistate state‐space model to estimate the parameters in Equations 1 and 2. Because we can detect the cause of death from predation by mice from our trail camera monitoring results (see Results), natural selection was estimated with the predation effects excluded. We did this by using two types for the “dead” state: “dead from predation by mice” and “dead from other causes” (Marescot et al., 2015; Schaub & Pradel, 2004). By dividing these, it was possible to determine which factors caused natural selection. This model was constructed by extending a previous multistate model (Ito & Konuma, 2020). In this model, we defined the state matrix with 12 states (Equation S1): (1) survival within the study site as a juvenile; (2) dead from other factors, for example, temperature, within the study site as a juvenile; (3) dead from the predation of mice within the study site as a juvenile; (4) survival within the study site as an adult; (5) dead from other factors within the study site as an adult; (6) dead from the predation of mice within the study site as an adult; (7) survival outside the study site as a juvenile; (8) dead from other factors outside the study site as a juvenile; (9) dead from the predation of mice outside the study site as a juvenile; (10) survival outside the study site as an adult; (11) dead from other factors outside the study site as an adult; and (12) dead from the predation of mice outside the study site as an adult.

In the matrix, we defined the survival rate of juveniles and adults, the predation rate from mice on juveniles and adults, the site fidelity rate, and the transition rate from juveniles to adults. The survival rate was applied to Equations 1 and 2 separately according to our purpose. The predation rate was assumed to be constant regardless of shell colour and month because when comparing the causes of mortality among marked snails, the cause of predation was not correlated with shell colour when we analyzed it using GLMM (see the Results). According to a previous study, the transition rate was assumed to fluctuate monthly, and the site fidelity rate was considered constant (Ito & Konuma, 2020). The transition rate was modeled with a random effects model.

In the observation matrix, we defined seven observation states (Equation S2): (1) first capture or recapture of a living juvenile snail; (2) recovery of a juvenile snail that died from causes other than predation; (3) recovery of a juvenile snail that died from predation; (4) first capture or recapture of a living adult snail; (5) recovery of an adult snail that died from causes other than predation; (6) recovery of an adult snail that died from predation; and (7) an undiscovered snail or unrecovered marked snail. The recapture rate was for live individuals in this matrix, and the recovery rate was for marked dead individuals. These parameters were defined as differing between survey months, although they were the same for juveniles and adults. In addition, these parameters could vary according to shell colour (Ito & Konuma, 2020). We modeled the effect of shell colour using a GLMM framework, in which the link function was a logit function. In addition, the same individual heterogeneity *ε_rp_*_,_ *_i_* was defined in these parameters. This heterogeneity parameter is normally distributed with a mean of 0 and variance $\sigma_{\mathrm{rp}}^{2}$. The probability models obtained from the state and observation matrices were defined with categorical distributions (Equations S3 and S4; Kery & Schaub, 2012).

### 2.5 Estimating parameters in the Bayesian model

We used Markov chain Monte Carlo (MCMC) simulations in the Bayesian framework to estimate the parameters in each model. To estimate and visualize natural selection in the model, we also used a noninformative prior distribution with the same parameters described in a previous study (Ito & Konuma, 2020). To avoid numerical instability and improve the mixing of each chain, we standardized the shell colour by the z‐score before using the MCMC (Gilks & Roberts, 1996). To obtain the posterior distribution of each parameter in our models, we generated three Markov chains using 2000 MCMC simulations that were thinned at a rate of 5 following the initial burn‐in of 1,000 iterations. In the model to visualize natural selection, 2,500 MCMC simulations were required. We confirmed the Gelman and Rubin statistics to be lower than 1.1 with all simulations (Gelman et al., 2011). A 95% Bayesian confidence interval (BCI) for all parameters was then used for the posterior distribution summary. For the MCMC simulations, we used pystan (https://github.com/stan‐dev/pystan) in Python 3.

## 3 RESULTS

**FIGURE 2:**
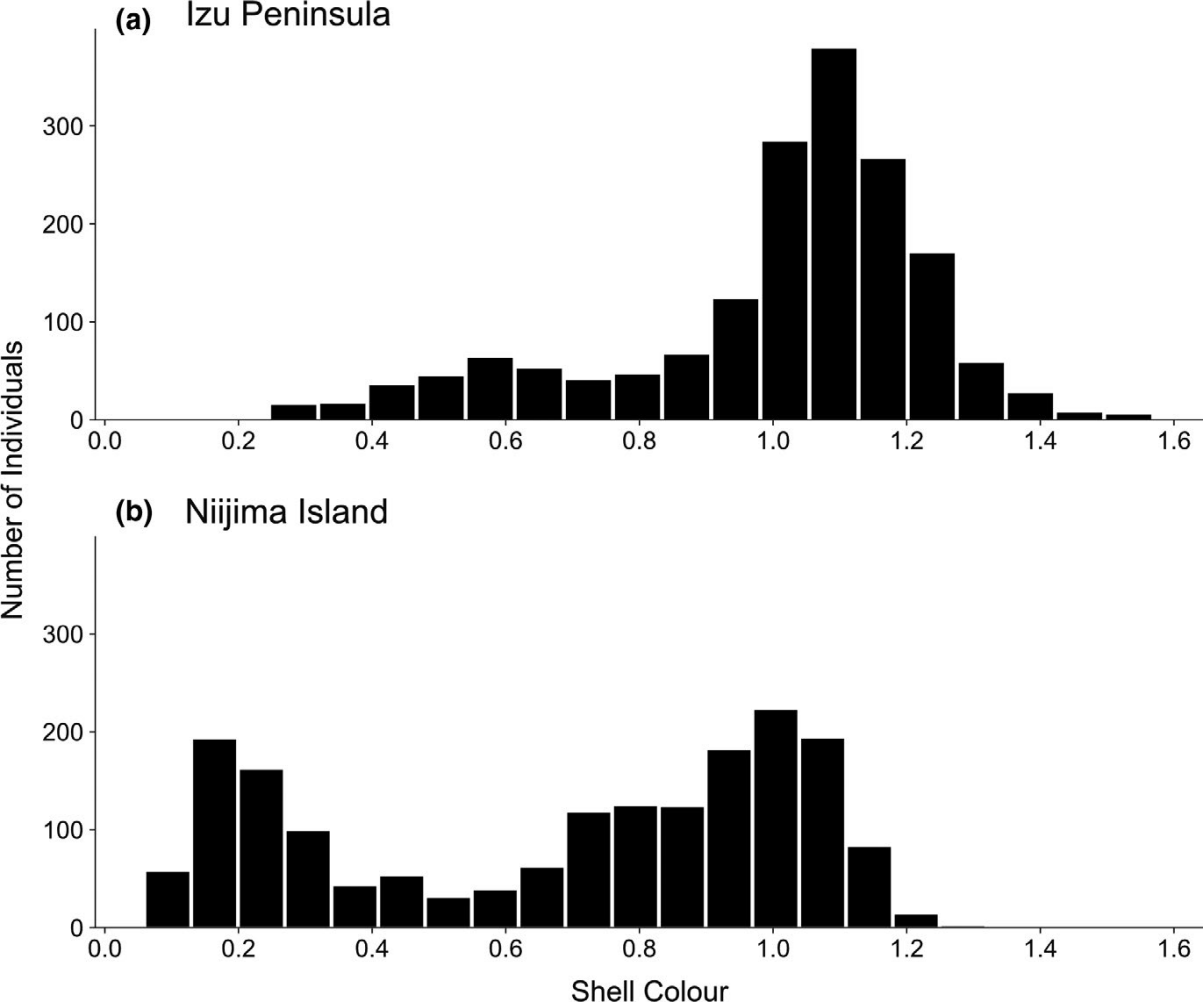
Frequency distribution of shell colour on marked snails in (a) Izu Peninsula and (b) Niijima Island (modified from Ito & Konuma, 2020). Shell colour was quantified using the luminance value *Y* using a photograph of the snail, with higher values representing brighter colours. In the Izu Peninsula, most of the snails had bright shells whereas in Niijima, snails with dark and bright shell colour existed with almost equal frequency

In the mark–recapture experiment conducted on the mainland, we marked and released 1,714 snails in total. We re‐encountered 337 of the snails released in the field (257 living and 80 dead). Some snails were recaptured more than once, with a maximum of 4 times. Most of the snails on the mainland had bright shells (Figure 2a), with a median of 1.066 and variance of 0.055. Snails with an intermediate shell colour also existed on the Izu Peninsula. In contrast, there was no bias toward bright shell colours on the island (Figure 2b); there, the median was 0.787, and the variance was 0.121. The medians and variances significantly differed (Wilcoxon rank‐sum test: *p* < .001; *F* test: *p* < .001).

**FIGURE 3:**
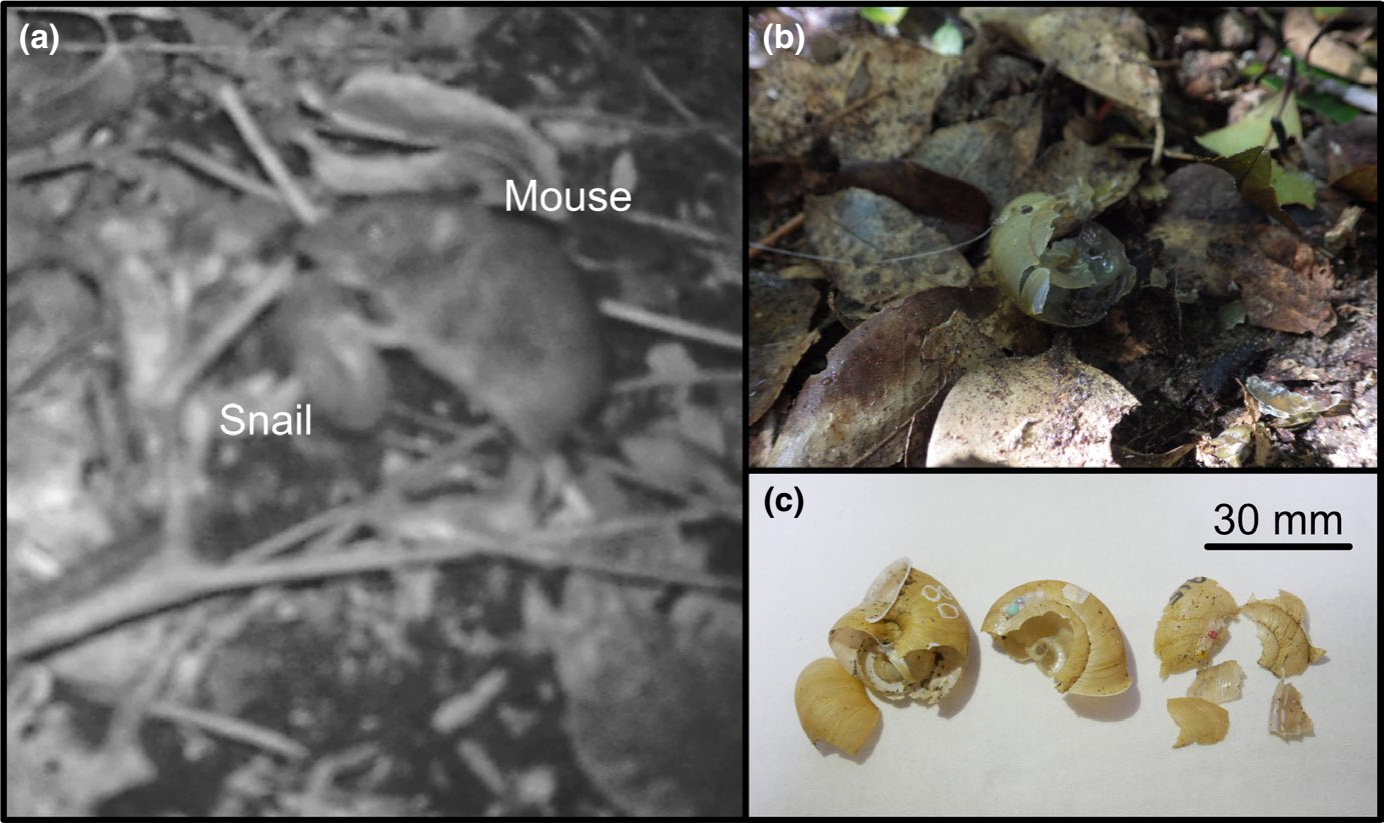
Images of preyed empty shells and the large Japanese field mouse *Apodemus speciosus*. (a) Preying on a live snail in front of a trail camera. (b) Shells left in place after the trail camera captured *A. speciosus* preying on snails. (c) Empty shells predated by *A. speciosus* found during the mark–recapture survey. The shells are characterized by cracked tops and shattered shells

Some recovered dead snails had evidence of shell crushing by predators (Figure 3). There were 37 such snails on the mainland and 8 on the island. More snails were subject to predation on the mainland than on the island (Figure S1; Fisher exact test, odds = 9.46, *p* < .001). In the GLMM analysis, the number of predated snails was not significantly correlated with the shell colour at either study site (Table 1; *z*‐value = 2.86, *p* > .05). However, the number of predated snails was correlated with growth stage on the mainland (Table 1). This result implies that predators attacked more adult than juvenile snails on the mainland. We conducted trail camera experiments to identify the predators and filmed 8 mammals, 4 birds, and 3 reptiles within the monitored snails' camera range (Table S1). Of these, only the large Japanese field mouse was attacking the snails (Figure 3c; Videos 1, 2, 3).

**TABLE 1:** Results of generalized linear mixed model on the association of cause of death in marked land snails (0: the others, 1: predated from mice) with shell colour and growth stage (juvenile and adult) on the Izu Peninsula and Niijima Island

	Fixed effect	Coefficient ± *SE*	*Z*‐value	*p*‐value
Izu Peninsula	Intercept	−1.24 ± 0.48	−2.56	<.05
	Shell colour	0.28 ± 0.43	0.64	.52
	**Growth stage**	**1.61 ± 0.56**	**2.86**	**<.001**
	Interaction	0.09 ± 0.55	0.16	.87
Niijima Island	Intercept	−3.17 ± 0.93	−3.41	<.001
	Shell colour	−0.15 ± 0.83	−0.18	.86
	Growth stage	0.49 ± 0.87	0.57	.57
	Interaction	0.55 ± 0.93	0.60	.55

Note

Bold values mean the *p*‐value was lower than 0.05.

**VIDEO 1:** A Scene where *Apodemus speciosus* attacks *Euhadra peliomphala simodae* tied to the ground by a 5 cm‐long string on the Izu Peninsula

**VIDEO 2:** A scene where *Apodemus speciosus* attacks *Euhadra peliomphala simodae* aestivated on the fallen leaf on the Izu Peninsula

**VIDEO 3:** A scene where *Apodemus speciosus* is preying on *Euhadra peliomphala simodae* tied to the ground by a 5 cm‐long string on the Niijima Island

Microhabitat use significantly differed between juveniles and adults on the mainland and island (Figure S2; Table S2; q‐value < 0.001). Microhabitat use also differed between the mainland and island (Figure S2; Table S2; q‐value < 0.001). On the mainland, microhabitat use did not correlate with shell colour for either juvenile or adult (Table 2; *z*‐value = −0.16, *p* = .87 for juveniles; *z*‐value = −1.41, *p* = .16 for adults). However, on the island, microhabitat correlated with shell colour for adults (Table 2; *z*‐value = −3.21, *p* < .01) but not for juveniles (*z*‐value = 0.12, *p* = .90).

**TABLE 2:** Results of generalized linear mixed model on the association of microhabitat use in marked land snails (0: ground, 1: arboreal) with shell colour on juvenile and adult on the Izu Peninsula and Niijima Island

	Stage	Fixed effect	Coefficient ± *SE*	*Z*‐value	*p*‐value
Izu Peninsula	Juvenile	Intercept	6.31 ± 2.49	2.51	<.05
		Shell colour	−0.04 ± 0.26	−0.16	.87
	Adult	Intercept	3.51 ± 0.78	4.51	<.001
		Shell colour	−0.24 ± 0.17	−1.41	.16
Niijima Island	Juvenile	Intercept	3.95 ± 0.63	6.31	<.001
		Shell colour	0.02 ± 0.15	0.12	.90
	Adult	Intercept	2.60 ± 0.53	4.88	<.001
		**Shell colour**	−**0.32 ± 0.09**	−**3.21**	**<.001**

Note

Bold values mean the *p*‐value was lower than 0.05.

**FIGURE 4:**
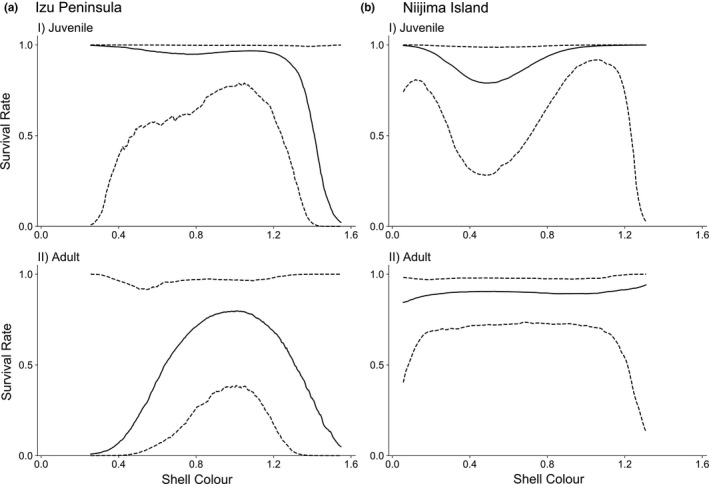
The relationship between survival rate and shell colour in (a) Izu Peninsula and (b) Niijima Island (modified from Ito & Konuma, 2020); (I) juvenile, (II) adult. This shape represents natural selection that worked on shell colour. In the Izu Peninsula, natural selection was estimated after excluding the effects of predation. The solid and dashed lines indicate the median and the 95% Bayesian confidence interval of the posterior distribution obtained from Markov chain Monte Carlo simulations, respectively

The survival rate in adults was 0.69 [0.32–0.94], while that in juveniles was 0.88 [0.58–0.98]. The P‐spline regression showed that the survival rates excluding predation effects were concave upward for the adults on the mainland (Figure 4a). In concordance with this, 94.2% of the *γ* values estimated in the simulation runs were negative for the adults (Table 3), although the 95% BCI for *γ* was [−2.47, 0.33]. In contrast, the P‐spline regression for the juveniles was not monomodal (Figure 4a), and the 95% BCI for *γ* included 0 (Table 3). The other parameters estimated from the Bayesian method are shown in Tables [Link], [Link], [Link].

**TABLE 3:** Estimated linear selection gradient *β* and nonlinear selection gradient *γ* and individual heterogeneity *σ* ^2^ using the Bayesian model on each growth stage

Stage	Parameter	Median	*SD*	95% BCI	
				2.5%	97.5%
Juvenile	*β*	−0.16	0.54	−1.35	0.73
	*γ*	−0.34	0.54	−1.35	0.73
	*σ* ^2^	3.68	3.63	0.21	12.9
Adult	*β*	0.01	0.66	−1.17	1.47
	γ	−1.05	0.70	−2.47	0.33
	σ^2^	9.77	3.32	3.11	14.8

Abbreviation: *SD*, standard deviation.

## 4 DISCUSSION

This study demonstrated that only the large Japanese field mouse, *A. speciosus*, preyed on the snails, but the predatory frequency was not correlated with shell colour (Figure 3; Table 1). In contrast, the predation frequency was higher on the mainland than on the island. Comparing the colour variation and microhabitat use of the snails between the mainland and island, we found that shell colour variation has diverged less on the mainland than on the island (Figure 2). The microhabitats used by the snails on the mainland were mainly on the ground, while the snails on the island used both arboreal and ground microhabitats. Furthermore, the mark–recapture experiments showed that the survival rate of bright‐shelled snails was highest on the mainland, while that on the island correlated with shell colour and had a dimorphic pattern (Figure 4a; Table 2). Hence, an ecological release due to predation release should have occurred, after which natural selection was modified, and the snails' shell colour diversified with expanding microhabitat use. In other words, shell colour diversity could have been induced by the ecological release of the island population.

The high predation pressure from field mice would be a primary factor in reducing survival rates on the mainland. Our trail camera experiments showed that field mice were the main predators of the snails. However, the number of predated snails was not correlated with their shell colour in the mark–recapture experiments. Like other field mice, these rodents do not recognize colour because they are colour blind, with only two cone types (Conway, 2007). Thus, the predation pressure from mice would occur regardless of shell colour (Rosin et al., 2011). Our results also implied that the field mouse is unlikely to prey on snails according to dark or bright shells. In other words, our results suggest that predation by field mice is a factor that only reduces snail survival.

Adult snails on the mainland mainly used ground microhabitats, while those on the island used both ground and arboreal microhabitats. The arboreal adult snails still need to come down from the trees to the ground to lay eggs (Inoue & Nakada, 1981). Our study demonstrated that adult snails have a lower survival rate than juvenile snails on the mainland. In addition, mainland adult snails were preyed on more than juvenile snails. Previous studies have reported that field mice are deficient at climbing trees and dwell on the ground (Imaizumi, 1978; Nishikata, 1981). Thus, the mainland adult snails might face a higher predation risk from the field mice on the ground. Furthermore, island snails experienced less predation than mainland snails. This suggests ecological release due to predation release on the island, which allowed for the expansion of microhabitat use to occur after migration to the island.

However, the predator on the mainland and island was the same, the large Japanese field mouse *A. speciosus*. One possible reason for this may be ecological evolution in the field mouse itself. A previous study suggested that the feeding behavior of the field mouse could have evolved after migration to the Izu Islands according to the island's environment due to the reduced ability to feed on walnuts in the Izu Islands (Takechi & Hayashi, 2012). Although a shift of feeding behavior to snails has not yet been revealed, the ecological evolution of the field mouse may have caused the difference in survival rates of the snails. A further examination of the feeding behavior of the field mouse on snails is needed to verify this explanation.

Predation could be an indirect driver of the persistence in shell colour monomorphism. High predation pressure can restrict a prey's habitat or microhabitat use to avoid predation (Lapiedra et al., 2018; Losos et al., 2004). The trait then fits the adaptive optimum according to the habitat or microhabitat use (Eklöv & Svanbäck, 2006). In other words, after migration to an island, a restricted trait can be diversified when an ecological release due to a release from high predation pressure occurs (Stroud & Losos, 2016). In the Izu Islands, a release from predation might have triggered the use of various habitats or microhabitats and been followed by phenotypic diversification.

That the highest survival rate was for bright‐shelled snails suggests weak stabilizing selection acts on shell colour from factors other than the predation of mice on the mainland. In contrast, our previous study demonstrated that disruptive selection worked on the shell colour on the islands (Ito & Konuma, 2020). In the previous study (Ito & Konuma, 2020), the disruptive selection estimated from the Bayesian method cannot exclude predation effects. However, the proportion of dead snails from predation was far lower on the island. Thus, it was suggested that the cause of the disruptive selection was also due to factors other than the predation of mice. In other words, both stabilizing and disruptive selection are caused by factors other than mice predation.

Strong disruptive selection acted only on the juveniles, while no selection acted on the adults on the island (Ito & Konuma, 2020). The reason why disruptive selection acted only on the island juveniles might be due to postzygotic isolation (Coughlan & Matute, 2020; Wade, 2002). Both dark and bright snails existed sympatrically, and the genetic relationships of the snails were close (Hayashi & Chiba, 2004). The dark and bright snails crossbreed, with the offspring showing an intermediate colour (Ito & Konuma, 2020; our unpubl. data). On the mainland, no selection acted on the juveniles, while stabilizing selection acted on the adults. The reason why stabilizing selection worked only on the adults might be related to their reproductive behavior. The snails on the mainland must change their microhabitat usage because they lay eggs on the ground (Inoue & Nakada, 1981). This expansion could have triggered the beginning of stabilizing selection on the mainland.

One hypothesis for the cause of natural selection is that shell colour may play a role in thermal regulation, as discussed in previous studies (Di Lellis et al., 2012; Ito & Konuma, 2020; Schweizer et al., 2019). For example, the brighter shell may help maintain an optimum body temperature when exposed to sunlight in open environments. In comparison, a darker shell could be advantageous when exposed to less sunlight in closed environments. Predation from birds is an alternative hypothesis, because bird predators promote shell colour diversification (Allen et al., 1988; Kraemer et al., 2019). For example, a brighter shell could provide camouflage in open habitat, while a darker shell would have the opposite effect. However, the birds captured by the trail cameras in our study did not show any predatory behavior, making this possibility less likely for this snail than the environmental hypothesis. This was the same to the case for the polymorphism in *Cepaea nemoralis* (Schilthuizen, 2013). As there is the possibility that a predator eats the shells whole or takes them away, we cannot completely rule out other predators as a cause of selection pressure. However, some mammals and birds were filmed in our trail camera experiments, and they did not prey on the snails. In any case, further field experiments are needed to elucidate the cause of the natural selection.

In conclusion, our findings suggest that stabilizing selection was working on the mainland. In contrast, disruptive selection acted on the shell colour on the island (Ito & Konuma, 2020), which had diversified from the ancient mainland population it derived from. Although it is still unknown what factors caused the shift in natural selection, the modification might result from ecological release due to a release from predation. These snails also inhabit other islands near Niijima Island, with shell colour patterns that differ from those on Niijima Island; intermediate‐coloured snails exist on some Izu islands (Hayashi & Chiba, 2004). The Izu Islands have different predator faunas and island‐specific predator–prey relationships (Hasegawa, 2003; Hasegawa & Moriguchi, 1989). Therefore, island‐specific relationships might induce differences in predation pressure and regulate the microhabitat usage and shell colour diversity on each island. In addition, recent studies have implied that microhabitat shifts due to predation release cause phenotypic diversification in a closed system such as a mainland–island system (Eklöv & Svanbäck, 2006; Lapiedra et al., 2018). Our study strongly supported this and further explains why we should consider the complex interplay of multiple factors when verifying phenotypic diversity.

## CONFLICT OF INTEREST

The authors have no conflict of interest to declare.

## AUTHOR CONTRIBUTIONS

**Shun Ito:** Conceptualization (equal); Data curation (lead); Formal analysis (lead); Investigation (lead); Methodology (lead); Project administration (equal); Validation (equal); Visualization (lead); Writing‐original draft (equal); Writing‐review & editing (equal). **Takahiro Hirano:** Conceptualization (supporting); Funding acquisition (supporting); Methodology (supporting); Writing‐review & editing (equal). **Satoshi Chiba:** Conceptualization (supporting); Funding acquisition (equal); Methodology (supporting); Project administration (equal); Writing‐review & editing (equal). **Junji Konuma:** Conceptualization (supporting); Funding acquisition (equal); Project administration (equal); Supervision (lead); Writing‐original draft (equal); Writing‐review & editing (equal).

## Supporting information

Fig S1

Fig S2

Table S1

Table S2

Table S3

Table S4

Table S5

Supplementary Material

## Data Availability

Source code and data used in the study are available from the Dryad (https://doi.org/10.5061/dryad.rr4xgxd8v). The stan codes used in mark–recapture are available from GitHub (https://github.com/sito7330i/articles/tree/master/Ito_et_al_2021).
