# Supplementary material for: Shell colour diversification induced by ecological release: A shift in natural selection after a migration event
Source: Ecol Evol. 2021 Oct 19;11(22):15534–44. doi: 10.1002/ece3.8080 (PMC8601913; doi:10.1002/ece3.8080)
Supplement: Supplementary file 1 — Fig S1 [file ECE3-11-15534-s008.docx]

**
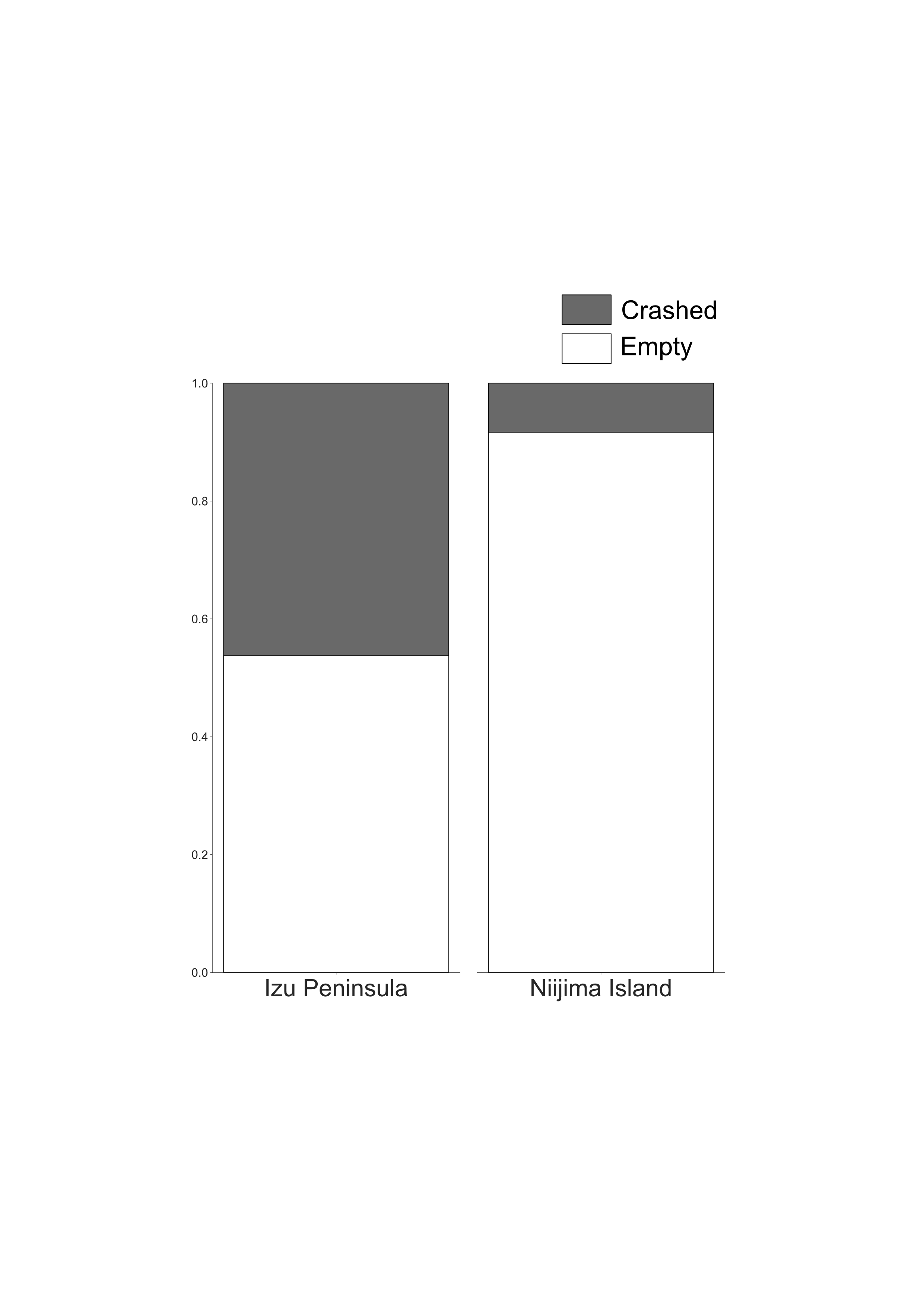
**

**Figure S1.** Proportion of dead cause of marked snails on the Izu Peninsula (left; mainland) and the Niijima Island (right; island). Dark-grey bars indicate crashed shells which were preyed on; white bars indicate empty shells which were dead from the other factor such as temperature and longevity.
