# Supplementary material for: Shell colour diversification induced by ecological release: A shift in natural selection after a migration event
Source: Ecol Evol. 2021 Oct 19;11(22):15534–44. doi: 10.1002/ece3.8080 (PMC8601913; doi:10.1002/ece3.8080)
Supplement: Supplementary file 2 — Fig S2 [file ECE3-11-15534-s006.docx]

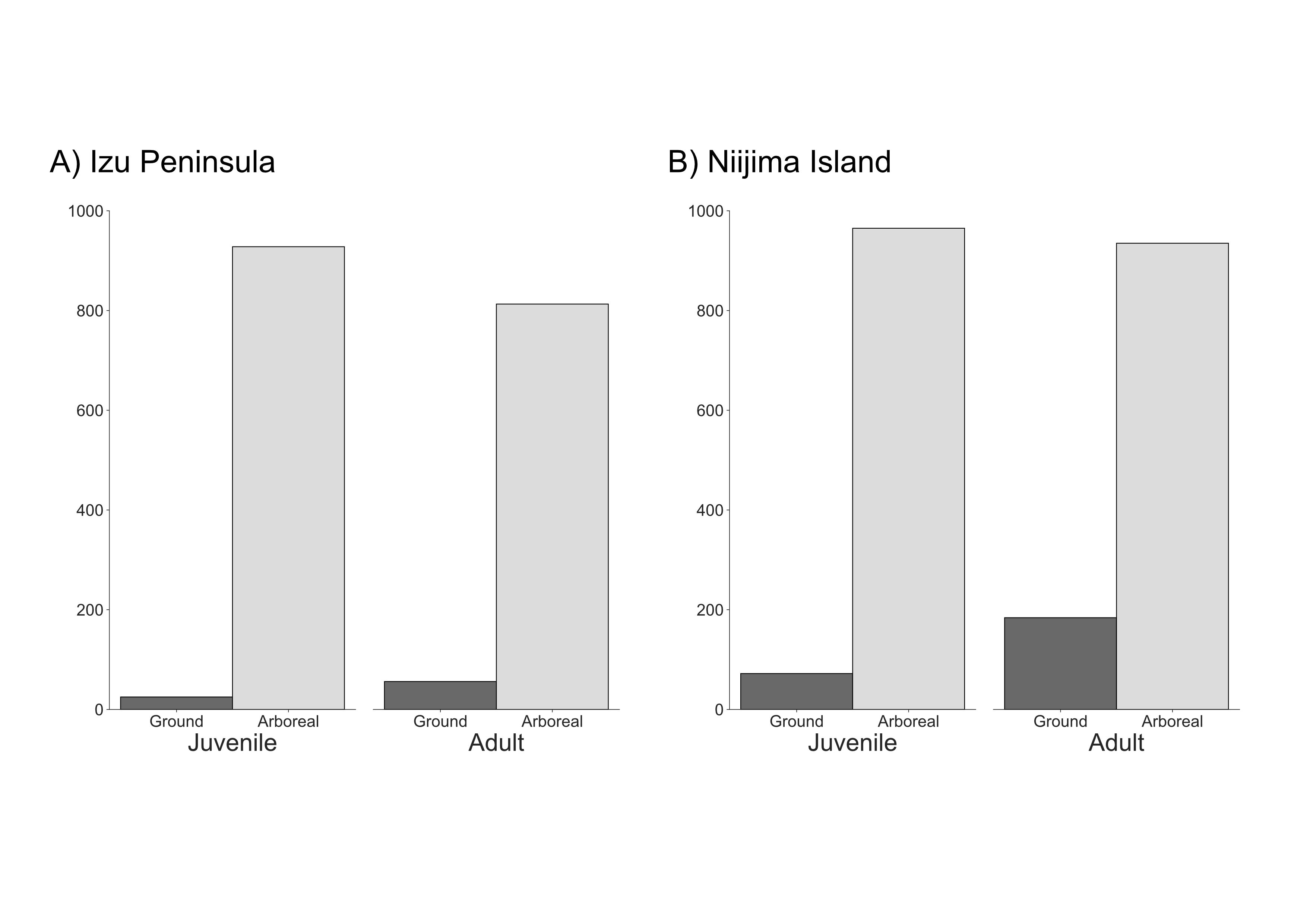


**Figure S2.** Proportion of microhabitat use of marked snails on the Izu Peninsula (A; mainland) and the Niijima Island (B; island). Dark-grey bars indicate ground; grey bars indicate arboreal.
