# Supplementary material for: Shell colour diversification induced by ecological release: A shift in natural selection after a migration event
Source: Ecol Evol. 2021 Oct 19;11(22):15534–44. doi: 10.1002/ece3.8080 (PMC8601913; doi:10.1002/ece3.8080)
Supplement: Supplementary file 3 — Table S1 [file ECE3-11-15534-s003.docx]

**Table S1.** The species which recognised in trail cameras on each population.

| Class | Species Names | Scientific Names | Izu Peninsula | Niijima Island |
| --- | --- | --- | --- | --- |
| Mammalia | Dsinezumi shrew | *Crocidura dsinezumi* | - | ○ |
|  | Large Japanese field mouse | *Apodemus speciosus* | ○ | ○ |
|  | Small Japanese field mouse | *A. argenteus* | ○ | - |
|  | Japanese hare | *Lepus brachyurus brachyurus* | ○ | - |
|  | Japanese badger | *Meles anakuma* | ○^※2^ | - |
|  | Wild boar | *Sus scrofa* | ○ | - |
|  | Japanese deer | *Cervus nippon aplodontus* | ○ | ○^※1^ |
|  | Domestic cat | *Felis silvestris catus* | - | ○ |
| Aves | Oriental turtle dove | *Streptopelia orientalis* | ○ | ○ |
|  | Japanese wood pigeon | *Columba janthina* | - | ○ |
|  | Olive-backed Pipit | *Anthus hodgsoni* | - | ○ |
|  | Red-flanked bluetail | *Tarsiger cyanurus* | ○ | - |
|  | Pale thrush | *Turdus pallidus* | ○ | ○ |
|  | White's Thrush | *Zoothera dauma* | - | ○ |
|  | Grey Bunting | *Emberiza variabilis* | ○ | - |
| Reptilia | Japanese four-lined rat snake | *Elaphe quadrivirgata* | - | ○ |
|  | Okada's blue-tailed skink | *Plestiodon latiscutatus* | - | ○ |
|  | Japanese common toad | *Bufo japonicus* | - | ○^※1^ |

○: The individuals were recognized. -: No individuals were recognised.

^※1^An alien species. ^※2^The species was confirmed at the line census although it did not appear on the camera.
