# Supplementary material for: Shell colour diversification induced by ecological release: A shift in natural selection after a migration event
Source: Ecol Evol. 2021 Oct 19;11(22):15534–44. doi: 10.1002/ece3.8080 (PMC8601913; doi:10.1002/ece3.8080)
Supplement: Supplementary file 4 — Table S2 [file ECE3-11-15534-s007.docx]

**Table S2.** Multiple comparisons on a proportion of microhabitat use between juvenile and adult in the mainland and an island by controlling false discovery rate. The q-values calculated from the p-value estimated from the Fisher exact test on each comparison. Bold values show that q-value lower than 0.05.

|  | | Izu Peninsula | | Niijima Island | |
| --- | --- | --- | --- | --- | --- |
|  |  | Juvenile | Adult | Juvenile | Adult |
| Izu Peninsula | Juvenile | – | – | – | – |
|  | Adult | **< 0.001** | – | – | – |
| Niijima Island | Juvenile | **< 0.001** | 0.71 | – | – |
|  | Adult | **< 0.001** | **< 0.001** | **< 0.001** | – |
