# Supplementary material for: Shell colour diversification induced by ecological release: A shift in natural selection after a migration event
Source: Ecol Evol. 2021 Oct 19;11(22):15534–44. doi: 10.1002/ece3.8080 (PMC8601913; doi:10.1002/ece3.8080)
Supplement: Supplementary file 5 — Table S3 [file ECE3-11-15534-s005.docx]

**Table S3.** The survival rate $S_{j}$ and monthly fluctuations $BJ_{t}$ of juvenile snails estimated from MCMC.

| Parameter | Median | SD | 95% BCI | |
| --- | --- | --- | --- | --- |
|  |  |  | 2.5% | 97.5% |
| $S_{j}$ | 0.88 | 0.11 | 0.58 | 0.98 |
| $BJ_{1}$ | 0.13 | 1.68 | -0.94 | 3.54 |
| $BJ_{2}$ | 0.84 | 1.45 | -1.32 | 4.36 |
| $BJ_{3}$ | 0.98 | 1.57 | -1.58 | 4.46 |
| $BJ_{4}$ | 1.38 | 1.48 | -0.81 | 4.72 |
| $BJ_{5}$ | 0.92 | 1.29 | -0.93 | 4.40 |
| $BJ_{6}$ | 0.40 | 1.0 | -1.51 | 2.55 |
| $BJ_{7}$ | 0.09 | 1.14 | -1.75 | 2.78 |
| $BJ_{8}$ | 1.44 | 1.56 | -0.75 | 5.32 |
| $BJ_{9}$ | -0.14 | 1.22 | -2.24 | 2.56 |
| $BJ_{10}$ | -4.98 | 1.67 | -8.56 | -2.18 |
| $BJ_{11}$ | -1.19 | 2.22 | -5.76 | 3.33 |
| $BJ_{12}$ | -0.59 | 2.06 | -4.92 | 3.19 |

SD: Standard Deviation
