# Supplementary material for: Shell colour diversification induced by ecological release: A shift in natural selection after a migration event
Source: Ecol Evol. 2021 Oct 19;11(22):15534–44. doi: 10.1002/ece3.8080 (PMC8601913; doi:10.1002/ece3.8080)
Supplement: Supplementary file 6 — Table S4 [file ECE3-11-15534-s002.docx]

**Table S4.** The survival rate $S_{a}$ and monthly fluctuations $BA_{t}$ of adult snails estimated from MCMC.

| Parameter | Median | SD | 95% BCI | |
| --- | --- | --- | --- | --- |
|  |  |  | 2.5% | 97.5% |
| $S_{a}$ | 0.69 | 0.16 | 0.32 | 0.94 |
| $BA_{1}$ | 1.03 | 1.71 | -1.89 | 4.89 |
| $BA_{2}$ | -0.01 | 1.53 | -2.81 | 3.42 |
| $BA_{3}$ | 0.62 | 1.43 | -1.71 | 4.14 |
| $BA_{4}$ | 0.78 | 1.34 | -1.33 | 4.0 |
| $BA_{5}$ | -0.56 | 1.16 | -2.59 | 2.04 |
| $BA_{6}$ | 0.01 | 1.32 | -2.06 | 3.33 |
| $BA_{7}$ | 1.08 | 1.41 | -0.13 | 5.69 |
| $BA_{8}$ | 1.36 | 1.3 | -0.86 | 4.41 |
| $BA_{9}$ | 0.05 | 1.01 | -1.95 | 2.06 |
| $BA_{10}$ | -2.38 | 1.0 | -4.33 | -0.44 |
| $BA_{11}$ | -0.79 | 1.98 | -4.46 | 2.98 |
| $BA_{12}$ | -3.14 | 2.28 | -6.87 | 1.61 |

SD: Standard Deviation
