# Supplementary material for: Shell colour diversification induced by ecological release: A shift in natural selection after a migration event
Source: Ecol Evol. 2021 Oct 19;11(22):15534–44. doi: 10.1002/ece3.8080 (PMC8601913; doi:10.1002/ece3.8080)
Supplement: Supplementary file 7 — Table S5 [file ECE3-11-15534-s004.docx]

**Table S5.** Predated rate $pred_{j}$ and $pred_{a}$, growth rate $g$, fidelity rate $F$, recapture rate $p$, recovery rate $r$, the coefficient of the recapture rate $\beta_{p}$ and the recovery rate $\beta_{r}$, individual heterogeneity $\sigma_{pr}^{2}$ of the recapture and the recovery rate, and monthly heterogeneity of recapture rate $\sigma_{r}$ and recovery rate $\sigma_{p}$ estimated from MCMC.

| Parameter | Median | SD | 95% BCI | |
| --- | --- | --- | --- | --- |
|  |  |  | 2.5% | 97.5% |
| $pred_{j}$ | 0.26 | 0.07 | 0.13 | 0.42 |
| $pred_{a}$ | 0.60 | 0.07 | 0.46 | 0.73 |
| $g$ | 0.03 | 0.04 | $2.5\times{10}^{-3}$ | 0.15 |
| $F$ | 0.87 | 0.04 | 0.8 | 0.97 |
| $p$ | 0.09 | 0.02 | 0.06 | 0.15 |
| $r$ | 0.01 | $6.4\times{10}^{-3}$ | $4.6\times{10}^{-3}$ | 0.02 |
| $\sigma_{g}$ | 8.05 | 3.6 | 2.3 | 14.63 |
| $\beta_{p}$ | -0.17 | 0.13 | -0.43 | 0.08 |
| $\beta_{r}$ | 0.34 | 0.14 | 0.07 | 0.66 |
| $\sigma_{r}$ | 0.48 | 1.26 | 0.07 | 4.61 |
| $\sigma_{p}$ | 0.32 | 0.36 | 0.04 | 1.38 |
| $\sigma_{rp}$ | 0.25 | 0.17 | 0.02 | 0.68 |

SD: Standard Deviationw
