# Supplementary material for: Shell colour diversification induced by ecological release: A shift in natural selection after a migration event
Source: Ecol Evol. 2021 Oct 19;11(22):15534–44. doi: 10.1002/ece3.8080 (PMC8601913; doi:10.1002/ece3.8080)
Supplement: Supplementary file 8 — Supplementary Material [file ECE3-11-15534-s001.docx]

**EQUATIONS OF STATE SPACE MODELLING**

State and observation matrix in multistate model were described following (Equations S1-S2):

$$\Omega=$$

$$\left[ \begin{matrix} \left( 1-g \right)\cdot F\cdot S_{j} & \left( 1-g \right)\cdot F\cdot(1-pred_{j})\cdot{(1-S}_{j}) & \left( 1-g \right)\cdot F\cdot pred_{j}\cdot{(1-S}_{j}) & g\cdot F\cdot S_{j} & g\cdot F\cdot(1-pred_{j})\cdot{(1-S}_{j}) & g\cdot F\cdot pred_{j}\cdot{(1-S}_{j}) & \left( 1-g \right)\cdot(1-F)\cdot S_{j} & \left( 1-g \right)\cdot(1-F)\cdot(1-pred_{j})\cdot{(1-S}_{j}) & \left( 1-g \right)\cdot(1-F)\cdot pred_{j}\cdot{(1-S}_{j}) & g\cdot(1-F)\cdot S_{j} & g\cdot(1-F)\cdot(1-pred_{j})\cdot{(1-S}_{j}) & g\cdot(1-F)\cdot pred_{j}\cdot{(1-S}_{j}) \\ 0 & 1 & 0 & 0 & 0 & 0 & 0 & 0 & 0 & 0 & 0 & 0 \\ 0 & 0 & 1 & 0 & 0 & 0 & 0 & 0 & 0 & 0 & 0 & 0 \\ 0 & 0 & 0 & {F\cdot S}_{a} & {F\cdot(1-pred_{j})\cdot(1-S}_{a}) & {F\cdot pred_{j}\cdot(1-S}_{a}) & 0 & 0 & 0 & (1-{F)\cdot S}_{a} & {(1-F)\cdot(1-pred_{j})\cdot(1-S}_{a}) & (1-{F)\cdot pred_{j}\cdot(1-S}_{a}) \\ 0 & 0 & 0 & 0 & 1 & 0 & 0 & 0 & 0 & 0 & 0 & 0 \\ 0 & 0 & 0 & 0 & 0 & 1 & 0 & 0 & 0 & 0 & 0 & 0 \\ 0 & 0 & 0 & 0 & 0 & 0 & \left( 1-g \right)\cdot S_{j} & \left( 1-g \right)\cdot(1-pred_{j})\cdot{(1-S}_{j}) & \left( 1-g \right)\cdot pred_{j}\cdot{(1-S}_{j}) & g\cdot S_{j} & g\cdot(1-pred_{j})\cdot{(1-S}_{j}) & g\cdot pred_{j}\cdot{(1-S}_{j}) \\ 0 & 0 & 0 & 0 & 0 & 0 & 0 & 1 & 0 & 0 & 0 & 0 \\ 0 & 0 & 0 & 0 & 0 & 0 & 0 & 0 & 1 & 0 & 0 & 0 \\ 0 & 0 & 0 & 0 & 0 & 0 & 0 & 0 & 0 & S_{a} & {(1-pred_{j})\cdot(1-S}_{a}) & {pred_{j}\cdot(1-S}_{a}) \\ 0 & 0 & 0 & 0 & 0 & 0 & 0 & 0 & 0 & 0 & 1 & 0 \\ 0 & 0 & 0 & 0 & 0 & 0 & 0 & 0 & 0 & 0 & 0 & 1 \end{matrix} \right]$$

$$(S1)$$

where $g$ means the transition rate from juvenile to adult, $F$ means site fidelity rate, $s_{j}$ and $s_{a}$ means survival rate of juvenile or adult, $pred_{j}$ and $pred_{a}$ means predated rate of juvenile or adult.

$$\begin{aligned} \Theta= \left[ \begin{matrix} p & 0 & 0 & 0 & 0 & 0 & 1-p \\ 0 & r & 0 & 0 & 0 & 0 & 1-r \\ 0 & 0 & r & 0 & 0 & 0 & 1-r \\ 0 & 0 & 0 & p & 0 & 0 & 1-p \\ 0 & 0 & 0 & 0 & r & 0 & 1-r \\ 0 & 0 & 0 & 0 & 0 & r & 1-r \\ 0 & 0 & 0 & 0 & 0 & 0 & 1 \\ 0 & 0 & 0 & 0 & 0 & 0 & 1 \\ 0 & 0 & 0 & 0 & 0 & 0 & 1 \\ 0 & 0 & 0 & 0 & 0 & 0 & 1 \\ 0 & 0 & 0 & 0 & 0 & 0 & 1 \\ 0 & 0 & 0 & 0 & 0 & 0 & 1 \end{matrix} \right] \#\left( S2 \right) \end{aligned}$$

where $p$ means recapture rate, $r$ means recovery rate.

The state-space equation of the multistate model was assumed by following (Equations S3-S4):

$$\begin{aligned} z_{i,f_{i}}=f\cdot s_{i} \end{aligned}$$

$$\begin{aligned} z_{i,t+1}|z_{i,t} \sim categorical\left( \Omega_{z_{i,t,1\ldots12,i,t}} \right) \#\left( S3 \right) \end{aligned}$$

$$\begin{aligned} y_{i,t}|z_{i,t} \sim categorical\left( \Theta_{z_{i,t,1\ldots7,i,t}} \right)\#\left( S4 \right) \end{aligned}$$

where $z_{i,t}$ means the state of $i$th individual at time $t$, $y_{i,t}$ is the observation of $i$th individual at time $t$, $z_{i,f_{i}}$ is the state of $i$th individual at first capture, and $s_{i}$ means $s_{j}$ or $s_{a}$ of $i$th individual according to growth stage.
